# Impact of trauma on society

**DOI:** 10.1007/s00068-025-02824-8

**Published:** 2025-03-26

**Authors:** Sascha Halvachizadeh, Diego Mariani, Roman Pfeifer

**Affiliations:** 1 Department for Traumatology, University Hospital Zurich, Zurich, Switzerland; 2 Asst Ovest Milanese, Chirurgia Generale E Urgenza, Legnano, Italy

**Keywords:** Polytrauma, Whitebook, ESTES

## Abstract

Trauma is the leading cause of death in the working population. The World Health Organisation (WHO) reports 4.4 million deaths annually due to unintentional or violence-related injuries; one in three of these deaths results from road traffic injuries (RTIs). For individuals aged 5–29 years, three of the top five causes of death are injury-related. Major trauma is the eighth leading cause of death across all age groups and the leading cause of death among children and young adults. The highest rates of trauma-related deaths are observed in low-income countries. Globally, men face twice the risk of dying from injuries as women, with approximately 75% of injury-related deaths resulting from trauma and RTIs.

## Current situation in Europe

**Fig. 1:**
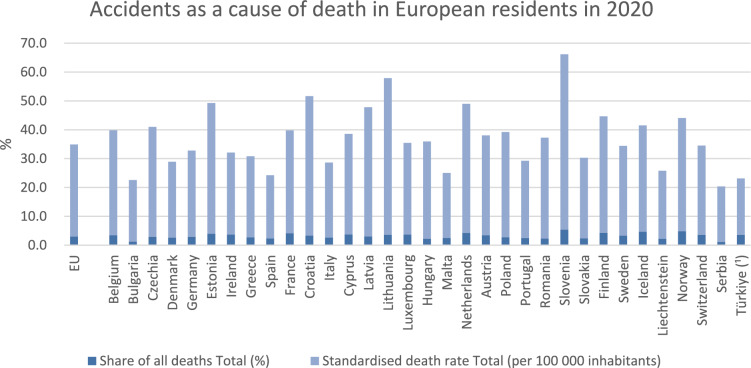
Accidents as a cause of death among European residents in 2020. (Publicly available data from Eurostat)

In 2020, 153,500 people in the European Union died from accidents, accounting for approximately 3.0% of all deaths (Fig. 1).

Trauma is the leading cause of mortality and disability-adjusted life-years (DALYs), particularly in Europeans aged 40 years and below. Following deaths due to malignant neoplasms of the trachea, bronchus, and lung (20.6% of all causes), injuries account for 20.2% of deaths in the European working population. Between 2011 and 2020, the overall mortality rate from RTIs declined from 7% to 4.9%. More than one-third of all deaths among individuals aged 15–19 years in Europe were trauma-related (Fig. 1).

**Fig. 2:**
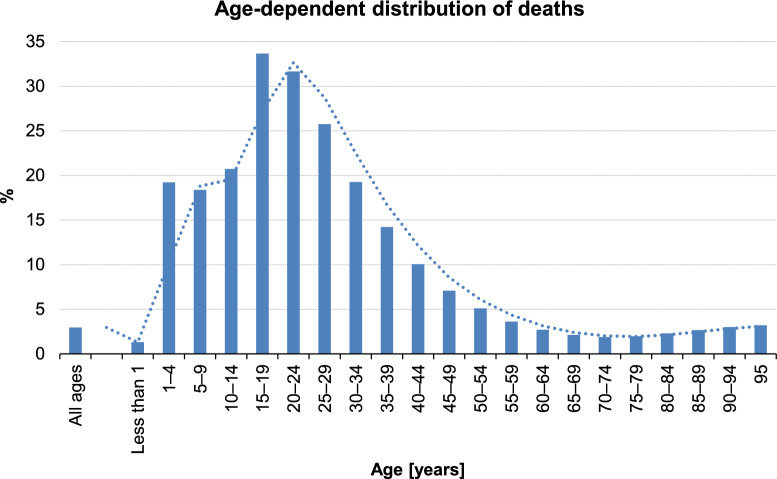
Trauma-related share of all deaths in the European population stratified by age, adopted from publicly available data from Eurostat

Depending on the country, 2.3% to 13.7% of European residents aged 15 years and older reported experiencing injuries at home or during leisure activities within a one-year period (Fig. 2).

**Fig. 3:**
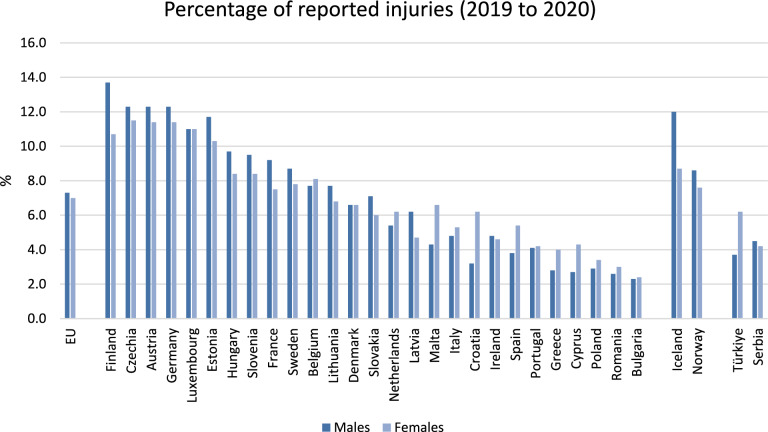
Male and Female Europeans aged 15 years and older reporting injuries within one year (publicly available data from Eurostat)

**Fig. 4:**
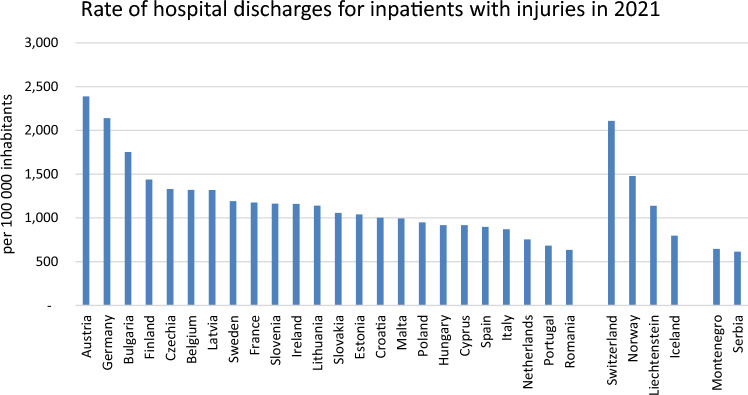
The rate of hospital discharges for inpatients with injuries in European countries in 2021 (publicly available data from Eurostat)

In 2021, the number of country-specific hospital discharges for inpatients with injuries ranged from 614 to 2389 per 100,000 inhabitants (Figs. 3, 4).

**Fig. 5:**
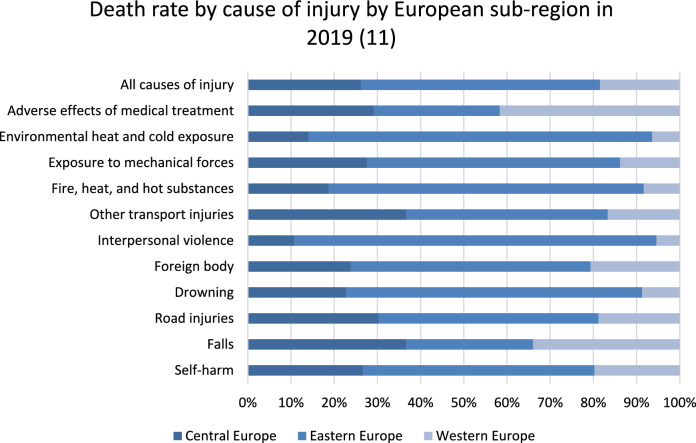
Death rate by cause of injury stratified by European sub-region in 2019

On average, the length of stay following injuries in Europe was 7.2 days (± 2.0) in 2016 and 7.0 days (± 1.7) in 2021. Patients with major fractures (e.g., femur fractures) experienced a decrease in length of stay by an average of 1.3 days (± 0.9) (see Fig. 5).

## Road traffic injuries (RTIs)

*Frequency*: RTIs are a leading cause of trauma in Europe, contributing to a substantial number of injuries and deaths. Factors including increased motorisation and road infrastructure play a role in their frequency.

**Fig. 6:**
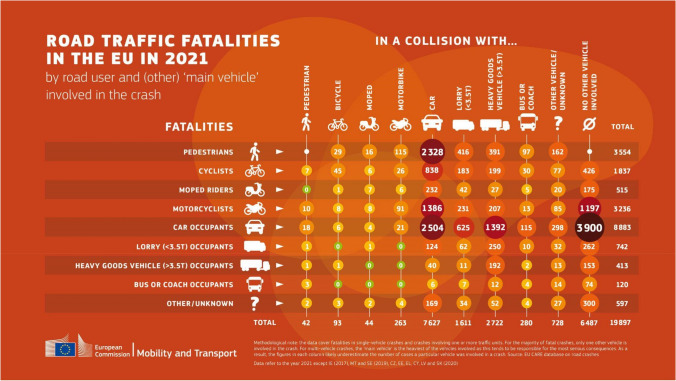
Road traffic Injuries in the EU

*Consequences*: These incidents have profound consequences for individuals and public health systems. Years of life lost (YLL) and years lived with disability (YLD) are metrics used to quantify the burden of disease. RTIs result in significant burdens of YLL and YLD, reflecting the impact of both fatalities and long-term disabilities. Fatalities and severe injuries often cause extensive YLL, while survivors may experience long-term disabilities, contributing to YLD (see Fig. 6).

## Self-harm and violence

*Frequency*: Interpersonal violence and self-inflicted injuries are major mechanisms of trauma. The average rate is 0.4 per 100,000 people (updated 2020 source, Small Arms Survey). These incidents vary across European countries, influenced by socio-economic factors, mental health issues, and societal circumstances.

*Consequences*: Interpersonal violence and self-inflicted injuries contribute significantly to both premature death and long-term disability, adding to YLL and YLD. The psychological impact on survivors is often profound.

## Unintentional injuries

Frequency: Unintentional injuries, including falls, burns, and other mechanisms, are common. Their frequency is influenced by environmental hazards and individual behaviours.

Consequences: The outcomes of unintentional injuries vary but often lead to YLD due to disabilities caused by injuries. Severe cases can also result in YLL.

## Reported global data

Studies have shown that trauma patients die at an early stage—either on site due to severe head injuries or within 48 h due to severe haemorrhage.

**Fig. 7:**
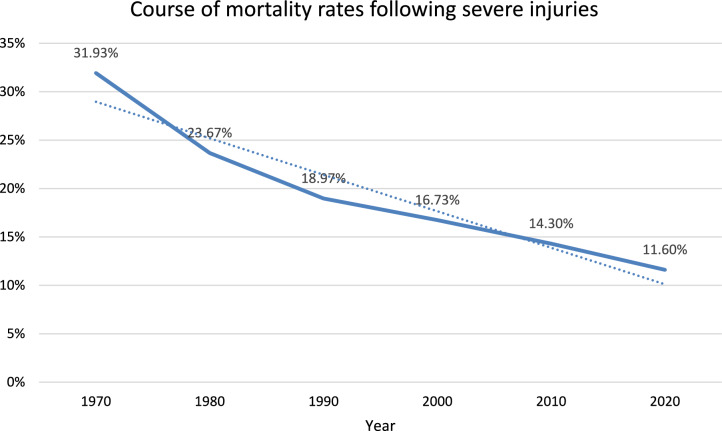
The global course of mean mortality rates following severe injuries over the past 5 decades

Improvement in trauma care is evident in the steady decline of mortality rates. Mortality rates dropped from 37 to 22% between the 1970s and 1990s. By the late 1990s, a mortality rate of 13.9% following severe injuries was reported (see fig. 7).

A summary of data regarding trauma related deaths published between 1980 and 2008 showed a decrease of haemorrhage-induced deaths from 25 to 18%. Traumatic brain injuries, however, are associated with an annual 2.5% increase in mortality rate (95% CI 1.9–3.0%).

## The burden of injury in Europe

**Fig. 8:**
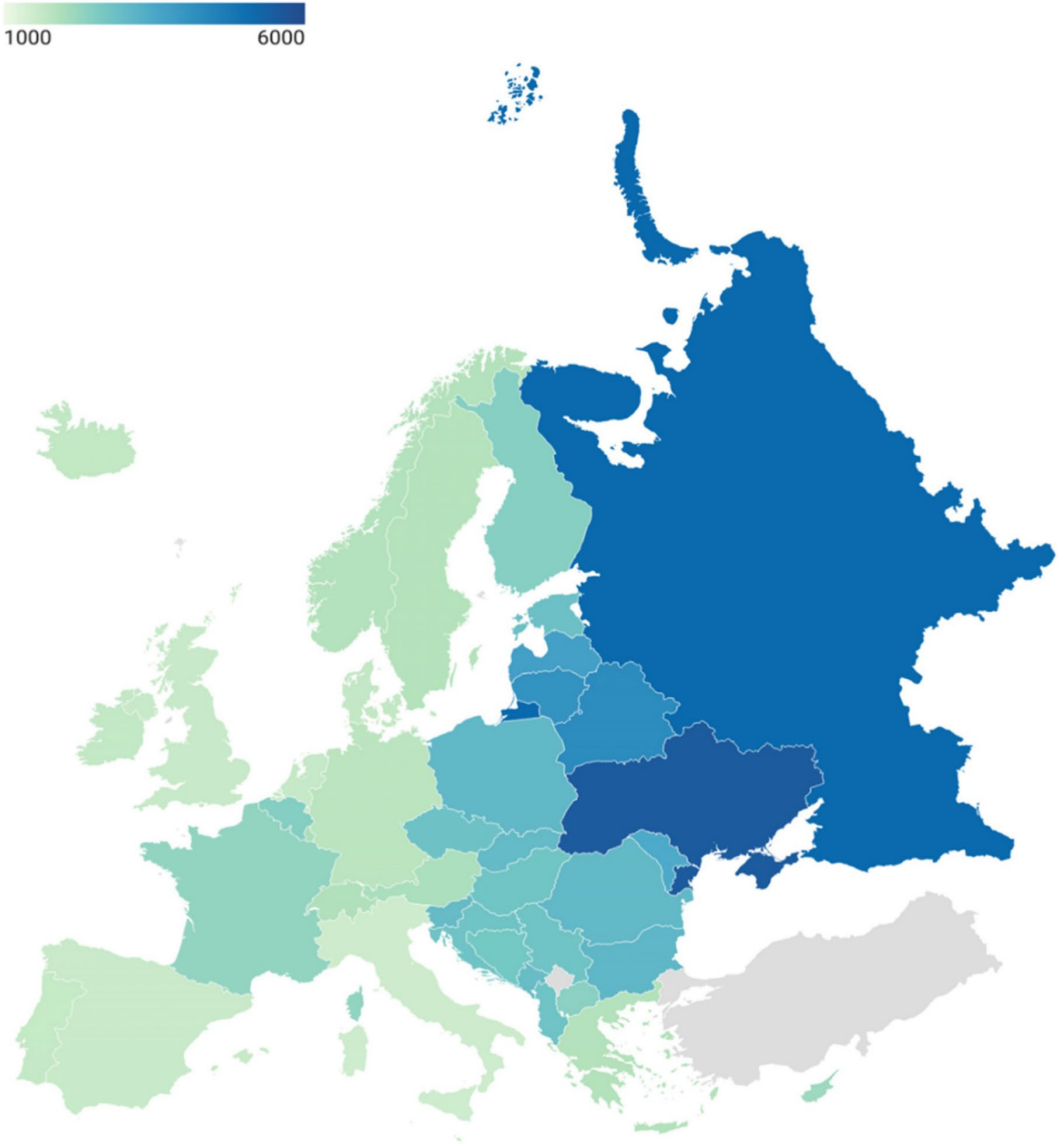
Age-standardised DALY rate of injury per 100,000 people per country, 2019. *Countries in grey indicate that they are not part of the GBD European sub-regions

In 2019, the disability-adjusted life years (DALYs) resulting from trauma ranged between 1781 and 5129 per 100 000 people in Europe (13). The highest DALYs were observed following RTIs, ranging from 1061.3 (95% CI 928.4–1,226.4) in Eastern Europe, to 648.2 (95% CI 551.5–754.0) in Central Europe, and the lowest, 314.6 (95% CI 291.2–341.2) in Western Europe. Trauma mortality rates followed the same patterns. Over the past decade, an increase of 0.5% in DALYs has been reported following falls in Western Europe, reaching 580.5 (95% CI 440.4–768.2) DALYs. Falls represent the leading cause of trauma-related DALYs in Europe, ranging from 580.5 (95% CI 440.4–768.2) in Western Europe to 712.9 (95% CI 566.8–924.1) in Eastern Europe. This gradient in DALYs across Europe may be attributed to a lack of unified trauma management structures and the absence of coordinated trauma systems (see fig. 8).

In Europe, the most significant injuries contributing to the highest YLD (Years Lived with Disability) are traumatic brain injuries, followed by spinal cord injuries and upper extremity amputations (Table 1).

**Table 1:** Ranking of injuries with the highest impact on YLD in Europe (14)

Injury	Years lived with disability (YLD)
Traumatic brain injury	86.8
Spinal cord injury	82.6
Amputation (upper extremity)	33.5
Hip fracture	30.4
Nerve injury	24.6
Amputation (lower extremity)	22.0
Femur shaft fracture	8.2
Lower leg fracture	1.0
Spine fracture	0.7
Elbow and forearm fracture	0.7

## Conclusion and needs for the future

Trauma remains the leading cause of mortality and DALYs, particularly among the European population aged 40 years and below. The improvement of trauma care in European countries often follows national initiatives; however, this leads to an unequal evolution of trauma care across Europe. Since advanced trauma systems improve survival rates and enhance quality of life, their implementation and maturation in each European country is of critical importance.

The trauma surgeon plays a vital role in this process, extending their involvement beyond surgical intervention to include the pre-hospital, hospital, and rehabilitation phases of trauma patient care. A Europe-wide consensus on trauma care could facilitate international communication and promote proportional improvements in national trauma systems.

## Bibliography

- Pfeifer R, Halvachizadeh S, Schick S, Sprengel K, Jensen KO, Teuben M, et al. Are pre-hospital trauma deaths preventable? A systematic literature review. World J Surg. 2019;43(10):2438–46.
- World Health Organization. Injuries and violence [Internet]. Geneva: World Health Organization; 2021 [cited 2023]. Available from: https://www.who.int/news-room/fact-sheets/detail/injuries-and-violence.
- World Health Organization. Global Status Report on Road Safety [Internet]. Geneva: World Health Organization; 2018 [cited 2023]. Available from: https://www.who.int/publications/i/item/9789241565684.
- Eurostat. Accidents and injuries statistics [Internet]. Luxembourg: Eurostat; 2020 [cited 2024]. Available from: https://ec.europa.eu/eurostat/statistics-explained/index.php?title=Accidents_and_injuries_statistics.
- Eurostat. Causes of death statistics [Internet]. Luxembourg: Eurostat; 2020 [cited 2024]. Available from: https://ec.europa.eu/eurostat/statistics-explained/index.php?title=Causes_of_death_statistics#Developments_from_2011-2020.
- Baker CC, Oppenheimer L, Stephens B, Lewis FR, Trunkey DD. Epidemiology of trauma deaths. Am J Surg. 1980;140(1):144–50.
- Evans JA, van Wessem KJ, McDougall D, Lee KA, Lyons T, Balogh ZJ. Epidemiology of traumatic deaths: comprehensive population-based assessment. World J Surg. 2010;34(1):158–63.
- Trunkey DD. Trauma. Accidental and intentional injuries account for more years of life lost in the U.S. than cancer and heart disease. Among the prescribed remedies are improved preventive efforts, speedier surgery and further research. Sci Am. 1983;249(2):28–35.
- Regel G, Lobenhoffer P, Grotz M, Pape HC, Lehmann U, Tscherne H. Treatment results of patients with multiple trauma: an analysis of 3406 cases treated between 1972 and 1991 at a German Level I Trauma Center. J Trauma. 1995;38(1):70–8.
- Nast-Kolb D, Aufmkolk M, Rucholtz S, Obertacke U, Waydhas C. Multiple organ failure still a major cause of morbidity but not mortality in blunt multiple trauma. J Trauma. 2001;51(5):835–41; discussion 41–2.
- Pfeifer R, Tarkin IS, Rocos B, Pape HC. Patterns of mortality and causes of death in polytrauma patients–has anything changed? Injury. 2009;40(9):907–11.
- van Breugel JMM, Niemeyer MJS, Houwert RM, Groenwold RHH, Leenen LPH, van Wessem KJP. Global changes in mortality rates in polytrauma patients admitted to the ICU-a systematic review. World J Emerg Surg. 2020;15(1):55.
- Haagsma JA, Charalampous P, Ariani F, Gallay A, Moesgaard Iburg K, Nena E, et al. The burden of injury in Central, Eastern, and Western European sub-region: a systematic analysis from the Global Burden of Disease 2019 Study. Arch Public Health. 2022;80(1):1–14.
- Polinder S, Meerding WJ, Mulder S, Petridou E, van Beeck E. Assessing the burden of injury in six European countries. Bull World Health Organ. 2007;85:27–34.
- Halvachizadeh S, Teuber H, Allemann F, Luidl AT, von Känel R, Zelle B, et al. Psychiatric outcome at least 20 years after trauma: A survey on the status of subjective general health and psychiatric symptoms with a focus on posttraumatic stress disorder. J Trauma Acute Care Surg. 2019;86(6):1027–32.
- Halvachizadeh S, Teuber H, Berk T, Allemann F, von Känel R, Zelle B, et al. Prevalence, injury-, and non-injury-related factors associated with anxiety and depression in polytrauma patients—A retrospective 20 year follow-up study. PLoS One. 2020;15(5):e0232678.
- Steel J, Youssef M, Pfeifer R, Ramirez JM, Probst C, Sellei R, et al. Health-related quality of life in patients with multiple injuries and traumatic brain injury 10 + years postinjury. J Trauma. 2010;69(3):523–30; discussion 30–1.
- Zelle BA, Marcantonio A, Tarkin IS. Functional long-term outcomes in polytrauma patients with orthopedic injuries. Damage control management in the polytrauma patient. 2010:439–52.
- Hietbrink F, Mohseni S, Mariani D, Naess PA, Rey-Valcárcel C, Biloslavo A, et al. What trauma patients need: the European dilemma. Eur J Trauma Emerg Surg. 2022.
- El-Menyar A, Mekkodathil A, Asim M, Consunji R, Strandvik G, Peralta R, et al. Maturation process and international accreditation of trauma system in a rapidly developing country. PLoS One. 2020;15(12):e0243658.
- MacKenzie EJ, Weir S, Rivara FP, Jurkovich GJ, Nathens AB, Wang W, et al. The value of trauma center care. J Trauma. 2010;69(1):1–10.
- International TAHP. International Health Facility Guidelines [Internet]. 2019 [cited 2024]. Available from: https://www.healthfacilityguidelines.com/.
- World Health Organization. Global Status Report on Road Safety 2018. Geneva: World Health Organization; 2019.
- World Health Organization. World report on road traffic injury prevention: summary. Geneva: World Health Organization; 2004 [cited 2025 Jan 12]. Available from: https://pesquisa.bvsalud.org/portal/resource/pt/bvs-2457.
- World Health Organization. World report on violence and health: summary. Geneva: World Health Organization; 2002.
- European Agency for Safety and Health at Work, Webster J, Brun E, Suarez A, Publikacji UEU, Europejska Agencja Bezpieczeństwa i Zdrowia w Pracy. Occupational safety and health in the wind energy sector. Luxembourg: Publications Office of the European Union; 2013.
- World Health Organization. The World Health Report 2007: A Safer Future: Global Public Health Security in the 21st Century. Geneva: World Health Organization; 2007.

## Data Availability

No datasets were generated or analysed during the current study.

